# Casein hydrolysate's effects on the fermentation properties, texture, and chemical characteristics and the bacterial microbiota of fermented glutinous rice dough

**DOI:** 10.3389/fmicb.2023.1292741

**Published:** 2023-11-22

**Authors:** Ling Guo, Biqi Liu, Yujun Jiang, Wei Zhang, Jing Han, Wenxuan Qu, Yuxi Han, Xinhuai Zhao, Xinyan Yang

**Affiliations:** ^1^Key Laboratory of Dairy Science, Ministry of Education, College of Food Science, Northeast Agricultural University, Harbin, China; ^2^Food Laboratory of Zhongyuan, Luohe, China; ^3^School of Biology and Food Engineering, Guangdong University of Petrochemical Technology, Maoming, China

**Keywords:** glutinous rice dough, casein hydrolysate, fermentation properties, bacterial composition, texture

## Abstract

To investigate how casein hydrolysate affected the physicochemical properties and microbiological diversity of the glutinous rice dough (natural fermentation and yeast fermentation), we analyzed its fermentation properties, carbohydrate, protein degradation, texture, and bacterial composition. According to the findings, casein hydrolysate increased the total LAB number, as well as organic acid content, in naturally fermented and yeast fermented glutinous rice dough by 3.59 and 8.19%, respectively, and reduced the fermentation time by at least 2 h. Meanwhile, casein hydrolysate enhanced the content of reducing sugars by 4.46 and 13.53% and increased protease activity by 29.9 and 27.7%. In addition, casein hydrolysate accelerated protein breakdown and regulated the hardness of the dough to improve the texture. Casein hydrolysate enriched the bacterial richness and diversity of dough. After adding casein hydrolysate, it promoted the growth of *Pediococcus, Enterococcus, Lactobacillus*, and *Streptococcus*. According to the Spearman correlation analysis, environmental factors (pH, lactic acid, acetic acid, reducing sugar content, and protease activity) exhibited the major driver for the abundance of bacterial species (Spearman correlation coefficient: −0.71 to 0.78). As a potential food additive, casein hydrolysate can improve the fermentation and quality of glutinous rice dough, increase consumer acceptance of cereal foods, and give consumers healthier options.

## 1 Introduction

Glutinous rice is a well-known traditional cereal food in China and even Southeast Asia (Li et al., 2018). As a result of its soft texture, higher stickiness, distinctive flavor, and nutritional value, it has been widely adapted to desserts, rice crackers, rice cakes, and rice dumplings (Maneerote et al., 2009; Qiu et al., 2020; Wang et al., 2022). Among them, fermented glutinous rice foods are one of the most distinctive foods, which have a substantial place in people's everyday diet, and are habitually associated with functional roles and health benefits. Unfortunately, fermented glutinous rice dough contain high levels of amylopectin, resulting in strong hardness, poor adaptability, low digestibility, and weak water holding ability (Eom et al., 2018; Meng and Kim, 2020b). Furthermore, it is challenging to accomplish large-scale industrial manufacturing due to their limited storage period (He et al., 2022). These disadvantages seriously affect the processing characteristics and quality of fermented glutinous rice dough. To overcome these defects, manufacturers and researchers evaluate the potential of food additives, such as hydrocolloids (e.g., xanthan gum, locust bean gum, hydroxypropyl cellulose, and hydroxypropyl methylcellulose) (Filipčev et al., 2021), enzymes (α-amylase, β-amylase, and xylanase) (Meng and Kim, 2020a), and emulsifiers (sucrose ester and sodium stearoyl lactylate) (Feng et al., 2021), in starchy foods with the intention of enhancing their nutritional value and quality.

In the past few years, protein hydrolysates have been increasingly chosen as an emerging bioactive component in functional food production because of their numerous functional properties and physiological health benefits (Rao et al., 2020). Among them, the non-toxic and inexpensive casein is more beneficial to consumers. Protein hydrolysate (CH) isolated from casein, which is a mixture of polypeptides, oligopeptides, and free amino acids, shorten the fermentation time, increase the lactic acid bacteria (LAB) population, and produce large quantities of bioactive peptides and functional amino acids (Bouhallab et al., 1993; Zhang et al., 2010; Nielsen et al., 2017). Previous studies have found that casein hydrolysate (CH) could influence the functional properties of wheat flour bread, including higher volume and lower firmness (Kenny et al., 2000). Additionally, researchers found that the addition of CH to wheat flour dough to produce bread showed favorable regulatory effects (Gani et al., 2015). However, there is currently no report focusing on the addition of CH as food additives in fermented glutinous rice dough. The effect of CH on the fermentation behavior, texture, bacterial microbiota, and other aspects of glutinous rice dough is still unknown.

Natural fermentation is the primary fermentation process used to produce fermented glutinous rice foods (Mishra et al., 2022). Meanwhile, such a fermentation method provides a better ecosystem for microorganisms, especially yeast and LAB (Siepmann et al., 2018). These microorganisms mainly contribute to improving the flavor, texture, nutritional value, and shelf-life characteristics of fermented cereal foods (Rezaei et al., 2016; Yu et al., 2019; He et al., 2022). However, it is difficult to commercialize these products as the fermentation time in dough is too long. Driven by the requirement of modern food industry, the most common industrial production method is rapid fermentation utilizing solely commercial yeast (Ma et al., 2021). Therefore, the influence of CH on fermentation behavior, textural and chemical features, and bacterial microbiota of glutinous rice dough was examined by natural fermentation and yeast fermentation. The aim of this study was to broaden the application potential of CH and explore a practical way to obtain a soft texture, favorable fermentation properties, and high nutritional value of glutinous rice dough and, more importantly, provide useful production guidance.

## 2 Materials and methods

### 2.1 Materials

The glutinous rice flour was obtained from the local supermarket (Harbin, Heilongjiang, China). *S. cerevisiae* was the product of Danbaoli Yeast Co., Ltd (Guangxi, China). Casein was provided by Sigma–Aldrich Co., Ltd (St. Louis, MO, USA). Alkaline protease (1 kU/g) was obtained from Novozymes Biotech Co., Ltd (Hunan, China). The other reagents, all of analytical grade, were supplied by Solarbio Co., Ltd (Beijing, China).

### 2.2 Preparation of dough and casein hydrolysate

Glutinous rice flour was combined in a 7:4 ratio with sterile distilled water (60°C) to make the dough (Huang et al., 2023). Additionally, 70 g of glutinous rice flour received 0.21 g of yeast for yeast fermentation. The mixture was subsequently fermented for 3 h (yeast fermentation) and 12 h (natural fermentation) at 37°C. CH was prepared using alkaline protease and casein according to a previous method (Zhao and Li, 2009). The degree of hydrolysis of CH was 13.92%. The dough was prepared in an aseptic environment and then stored at 4°C for further analysis. The samples with and without yeast at the 0 h of fermentation were named as DY and DN, respectively. The fermented samples with and without yeast were, respectively, named as FDY and FDN. FDY and FDN with an additional 1% (W/W) of CH were named as FDYH and FDNH, respectively.

### 2.3 Determination of pH value

The pH values of the samples were determined using the method of Gong et al. (2020). The samples were diluted 10 times with sterile distilled water, and then, the pH values of different fermentation time were measured with a pH meter (model 61181, YIERYI Co., Ltd, Shenzhen, China).

### 2.4 The colony counting of lactic acid bacteria

Based on the technique described by Zhang et al. (2021), the total number of LAB colonies was calculated. Following the homogenization of all samples with a sterile NaCl solution (0.9%, w/v), 10-fold serial dilution was carried out. For 10 times dilution series (up to 10^−6^), a Petri dish containing 20 ml of ready MRS agar (pH 6.5) and samples that had been diluted to the necessary amount was placed in the incubator for 48 h at 37°C. The colonies between 30 and 300 on each Petri dish were counted as the total number of LAB colonies.

### 2.5 Determination of the contents of lactic acid and acetic acid

The contents of lactic acid and acetic acid were evaluated by high-performance liquid chromatography (HPLC) (Waters 2695, Jingkerida Technology Co., Ltd., Beijing, China). A total of 2.5 g of the sample was homogenized with 25 ml H_2_SO_4_ (5 mmoL/L) for 1 min and then centrifuged at 4°C (6,000 *g*) for 10 min. A 0.22-μm filter membrane was used to filter the supernatant. The chromatographic conditions were executed based on the report by Sánchez (Sánchez et al., 2004). The injection volume is 10-μl. The Aminex HPX-87H column (300 × 7.8 mm, Bio-Rad, USA) was used for separation. The mobile phase was H_2_SO_4_ (5 mmol/L), and the flow rate was 0.5 ml/min at 30°C. A ultraviolet detector was used to measure the contents of lactic acid and acetic acid at 210 nm.

### 2.6 Determination of reducing sugar content and protein degradation

The 3, 5-dinitrosalicylic acid (DNS) technique was used to determine the amount of reducing sugar present in glutinous rice dough samples (Shi et al., 2021). The pH value was corrected to 8.2 by adding 1 mol/L of NaOH after the dough samples were dissolved in distilled water (1: 9, w/v). The supernatant obtained by stirring (45°C, 40 min) and centrifuging (1,698 *g*, 10 min) was mixed with DNS reagent [1: 2 (v/v)]. It was then combined with DNS reagent [1: 2 (v/v)], boiled for 5 min, and allowed to cool at room temperature. A UV spectrophotometer (UV 2996, Shanghai, China) was used to monitor the absorbance value at 610 nm.

The SDS-PAGE procedure was carried out as Gong et al. (2021) reported, with the appropriate changes. The protein fraction was extracted using the solution containing 50 mmol/L sodium phosphate (pH 6.9), 1 mmol/L EDTA, and 1% SDS. The supernatant obtained by stirring (25°C, 1 h) and centrifuging (10,000 *g*, 20 min) was the whole protein fraction. Subsequently, the supernatant was boiled in a water bath for 5 min. The gel slab contained both the separating gel (12%) and concentrated gel (5%). A 15-μl aliquot of the supernatants was loaded and subjected to electrophoresis at 120 V. The gels were stained with Coomassie Brilliant Blue R-250 (0.1%, w/v) and detained with a solution containing methanol (24%, v/v) and acetic acid (7%, v/v). The image was taken by a GelDoc-It Imaging System (UVP, Hamamatsu, Japan).

### 2.7 Determination of protease activity

The method of evaluating the protease activity in glutinous rice dough samples was described by Senyay-Oncel et al. (2014). Dough samples were dissolved in phosphate buffer (1: 10, w/v), stirred, and then centrifuged (5,000 *g*, 10 min). At 40°C for 10 min, the supernatant (1 ml) was incubated with 1% casein solution (1 ml). The addition of 2 ml of trichloroacetic acid terminated the reaction. After centrifuging (5,000 *g*, 8 min) the samples, 5 ml of Na_2_CO_3_ (0.5 mol/L) solution and 1 ml of Folin–Ciocalteu were added to 1 ml of the supernatant. The reaction mixture was let to stand for 30 min at room temperature, during which time the absorbance value was measured at 660 nm. The amount of tyrosine released from casein per minute at 40°C and pH 3.0 was considered to be 1 mg of protease activity.

### 2.8 Textural and microstructural measurements

The texture profile analysis of dough slices (15 mm thickness) was investigated using a texture analyzer (TA-XT plus texture analyzer, Stable Micro Systems Co., Ltd., England) with a cylinder probe P/0.5S (Lazaridou et al., 2018). Pre-test, test, and post-test speeds were 2.0, 1.0, and 1.0 mm/s, respectively. The applied deformation was 60%, and the text gap was 5 s.

The images of scanning electron microscope (SEM) of the samples were investigated based on Gong's description (Gong et al., 2020). After being fixed in 3% pentanediol, the samples (2 g) were washed three times with phosphate buffer (0.1 mol/L). Successively, the samples were fixed in 1% osmium tetroxide, washed again, and dehydrated using 30, 50, 70, 90, and 100% ethanol. The supernatants were freeze-dried (−80°C), ruptured, coated with gold, and then used for the following adherence. The moisture of handled samples was observed by a scanning electron microscope S-3400N (Hitachi High-Tech Co. Ltd., Tokyo, Japan).

### 2.9 High-throughput sequencing and sequence analysis

Total genome DNA was extracted using a TIANamp Stool DNA Kit, according to the manufacturer's instructions (cat#DP302-02, Tiangen, China). DNA was amplified with the 515F (5′-GTGCCAGCMGCCGCGGTAA-3′) and 806R (5′-GGACTACHVGGGTWTCTAAT-3′) for the V4 hypervariable regions of 16S rRNA gene. The DNA was denatured at 98°C for 30 s. Twenty-seven cycles consist of annealing at 50°C for 30 s, elongation at 72°C for 30 s, and the final denaturation at 72°C for 5 min (Zhang and Wang, 2017). PCR products were detected by electrophoresis on agarose gel (2%) and purified by GeneJET Gel Extraction Kit (Thermo Fisher Scientific, New York, USA). After quantification of DNA concentration using Qubit, all PCR products were analyzed by high-throughput sequencing using Ion S5^TM^XL (Thermo Fisher Scientific, Shanghai, China). Raw reads were quality filtered using QIIME software (version 1.9.1). Sequences were assigned to the same operational taxonomic units (OTUs) at 97% similarity using UPARSE software (version 7.0.1001). The alpha diversity indices, including Shannon, Simpson, Chao 1, and Ace, were analyzed for bacterial diversity in the sample. The bacterial composition of samples was evaluated using principal component analysis (PCA). To determine the correlation between the bacterial composition of samples and multiple physicochemical properties, the Spearman correlation analysis was carried out.

### 2.10 Statistical analysis

Each sample was measured or tested at least three times independently. Using SPSS 22.0 program (SPSS Inc., Chicago, IL, USA), one-way analysis of variance (ANOVA) test and Duncan's multiple-range tests were conducted. Origin Pro 2022 (Origin Lab Corporation, Northampton, MA, USA) was used to draw the figures.

## 3 Results and discussion

### 3.1 Effects of CH on fermentation behaviors

The changes in the pH and total LAB colony counts during the fermentation of the samples are shown in Table 1. Acidification is an important feature of dough fermentation. As the fermentation time increased, the pH of all samples tended to decrease. The pH of FDNH and FDN was not clearly different at the initial stage of fermentation. FDNH had a pH of 6.28 after 6 h of fermentation, which was significantly lower than that of FDN (6.33, *P* < 0.05). After 12 h of fermentation, the addition of CH caused a 6.4% decrease in the maximum pH. During the yeast fermentation, the pH of FDYH was significantly lower than that of FDY (*P* < 0.05). In addition, there was a trend toward an increase in the total number of LAB in samples. Compared with the fermentation samples without CH, the total number of LAB colonies in FDNH and FDYH increased by 3.59 and 8.19%, respectively, at 12 and 3 h of fermentation. Regarding the organic acid contents, the addition of CH also had a clear impact on all samples. The present results (Table 1) indicated a steady increase of the two acids from the beginning of fermentation. The sample prepared with CH (FDNH and FDYH) showed the higher organic acid contents compared with the other samples until the end of fermentation. Overall, changes in pH value, organic acid contents, and the number of LAB colonies are correlated with each other. A similar situation has been observed in a previous study using shrimp protein hydrolysates (Karimi et al., 2020). *Lactococcus lactis* can grow twice as fast when exposed to polypeptides having a molecular weight of under 3 kDa, according to the reports of CH (Bouhallab et al., 1993). The pH drop during fermentation attributed to the rapid production of organic acids by the LAB in the dough (Mousavi and Mousavi, 2019). Protein hydrolysates provide the nitrogen source for LAB and thus enhance their growth and metabolism (Lucas et al., 2004). After the addition of CH, the number of LAB colonies increased and the metabolic activity improved; meanwhile, organic acids were also produced, which ultimately decreased the pH and formed an acidic environment. As expected, the addition of CH to glutinous rice dough led to the improvement of dough fermentation properties.

**Table 1:** pH, total number of LAB colonies (log CFU/g) and lactic acid and acetic acid contents of samples at various fermentation stages.

**Item**	**Samples**	**Fermentation time (natural fermentation)/h**						
		**0**	**2**	**4**	**6**	**8**	**10**	**12**
pH	SDN	6.40 ± 0.01^a, A^	6.31 ± 0.01^b, B^	6.28 ± 0.01^b, B^	6.28 ± 0.00^b, B^	6.25 ± 0.01^a, B^	6.15 ± 0.00^a, C^	5.95 ± 0.05^a, D^
	SDNH	6.43 ± 0.03^a, A^	6.34 ± 0.01^a, B^	6.34 ± 0.01^a, B^	6.33 ± 0.02^a, B^	6.01 ± 0.01^b, C^	5.98 ± 0.01^b, C^	5.71 ± 0.01^b, D^
LAB	SDN	4.52 ± 0.02^b, F^	5.31 ± 0.03^a, E^	5.86 ± 0.09^a, D^	5.97 ± 0.06^b, D^	7.18 ± 0.03^b, C^	8.63 ± 0.05^b, B^	9.18 ± 0.02^b, A^
	SDNH	4.46 ± 0.02^a, G^	5.35 ± 0.04^a, F^	5.89 ± 0.09^a, E^	6.15 ± 0.07^a, D^	7.78 ± 0.05^a, C^	8.94 ± 0.02^a, B^	9.51 ± 0.03^a, A^
Lactic acid	SDN	0.00 ± 0.00^b, F^	0.02 ± 0.01^a, EF^	0.05 ± 0.02^a, DE^	0.07 ± 0.01^b, D^	0.14 ± 0.01^b, C^	0.23 ± 0.01^a, B^	0.29 ± 0.02^a, A^
	SDNH	0.01 ± 0.00^a, F^	0.04 ± 0.01^a, EF^	0.08 ± 0.02^a, DE^	0.12 ± 0.01^a, D^	0.18 ± 0.02^a, C^	0.25 ± 0.02^a, B^	0.32 ± 0.01^a, A^
Acetic acid	SDN	0.02 ± 0.00^a, E^	0.03 ± 0.00^b, E^	0.07 ± 0.02^a, DE^	0.10 ± 0.02^a, D^	0.17 ± 0.02^b, C^	0.27 ± 0.02^b, B^	0.33 ± 0.02^b, A^
	SDNH	0.01 ± 0.00^b, F^	0.05 ± 0.00^a, EF^	0.08 ± 0.02^a, DE^	0.12 ± 0.01^a, D^	0.28 ± 0.02^a, C^	0.39 ± 0.02^a, B^	0.47 ± 0.02^a, A^
**Item**	**Samples**	**Fermentation time (yeast fermentation)/h**						
		**0**	**0.5**	**1**	**1.5**	**2**	**2.5**	**3**
pH	SDY	6.54 ± 0.01^a, A^	6.52 ± 0.01^a, A^	6.33 ± 0.01^a, B^	6.31 ± 0.01^a, B^	6.24 ± 0.02^a, C^	6.23 ± 0.01^a, C^	6.15 ± 0.03^a, D^
	SDYH	6.41 ± 0.01^b, A^	6.33 ± 0.01^b, B^	6.15 ± 0.01^b, C^	6.12 ± 0.01^b, C^	6.06 ± 0.01^b, D^	5.99 ± 0.01^b, E^	5.93 ± 0.03^b, F^
LAB	SDY	2.55 ± 0.15^a, E^	3.85 ± 0.07^a, D^	4.12 ± 0.14^b, CD^	4.48 ± 0.27^a, C^	5.13 ± 0.11^b, B^	5.39 ± 0.07^b, AB^	5.62 ± 0.01^b, A^
	SDYH	2.59± 0.05^a, D^	2.59 ± 0.05^b, D^	4.53 ± 0.04^a, C^	4.80 ± 0.23^a, C^	5.35 ± 0.04^a, B^	5.86 ± 0.03^a, A^	6.08 ± 0.04^a, A^
Lactic acid	SDY	0.00 ± 0.00^a, E^	0.00 ± 0.00^a, E^	0.02 ± 0.00^a, D^	0.05 ± 0.01^a, C^	0.07 ± 0.00^b, B^	0.08 ± 0.00^b, AB^	0.09 ± 0.00^b, A^
	SDYH	0.00 ± 0.00^a, G^	0.00 ± 0.00^a, F^	0.02 ± 0.00^a, E^	0.06 ± 0.00^a, D^	0.08 ± 0.00^a, C^	0.09 ± 0.00^a, B^	0.11 ± 0.00^a, A^
Acetic acid	SDY	0.00 ± 0.00^a, G^	0.09 ± 0.00^b, F^	0.14 ± 0.00^b, E^	0.17 ± 0.01^b, D^	0.21 ± 0.00^b, C^	0.27 ± 0.00^b, B^	0.32 ± 0.00^b, A^
	SDYH	0.00 ± 0.00^a, G^	0.11 ± 0.00^a, F^	0.15 ± 0.00^a, E^	0.20 ± 0.00^a, D^	0.27 ± 0.00^a, C^	0.33 ± 0.00^a, B^	0.35 ± 0.00^a, A^

Values are expressed as means ± standard deviation (n = 3). Different lowercase (or uppercase) letters in the same column (or row) indicated significant differences between groups (P < 0.05).

Shortening fermentation time have advantages not only for facilitating productivity but also for controlling large-scale commercial production of dough. When the pH of samples (FDN and FDNH, FDY and FDYH) decreased to the same value during the whole fermentation period, the dough samples with CH required a shorter fermentation time. In detail, FDNY and FDYH's fermentation time was at least 2 h faster than that of FDN and FDY, respectively. CH could be regarded as having the functional potential to improve fermentation efficiency and shorten fermentation time. Similarly, the dough with amaranth selective protein hydrolysates had a better fermentation ability and lower pH value compared with the ordinary dough, which also verified that protein hydrolysates regulated the fermentation time (Karimi et al., 2021). The short fermentation time indicated a possible application of CH in the industrial bread market. Therefore, investigation of the effects and action mode of protein hydrolysates on the fermentation in dough is of high importance.

### 3.2 Effects of CH on reducing sugar

**Figure 1:**
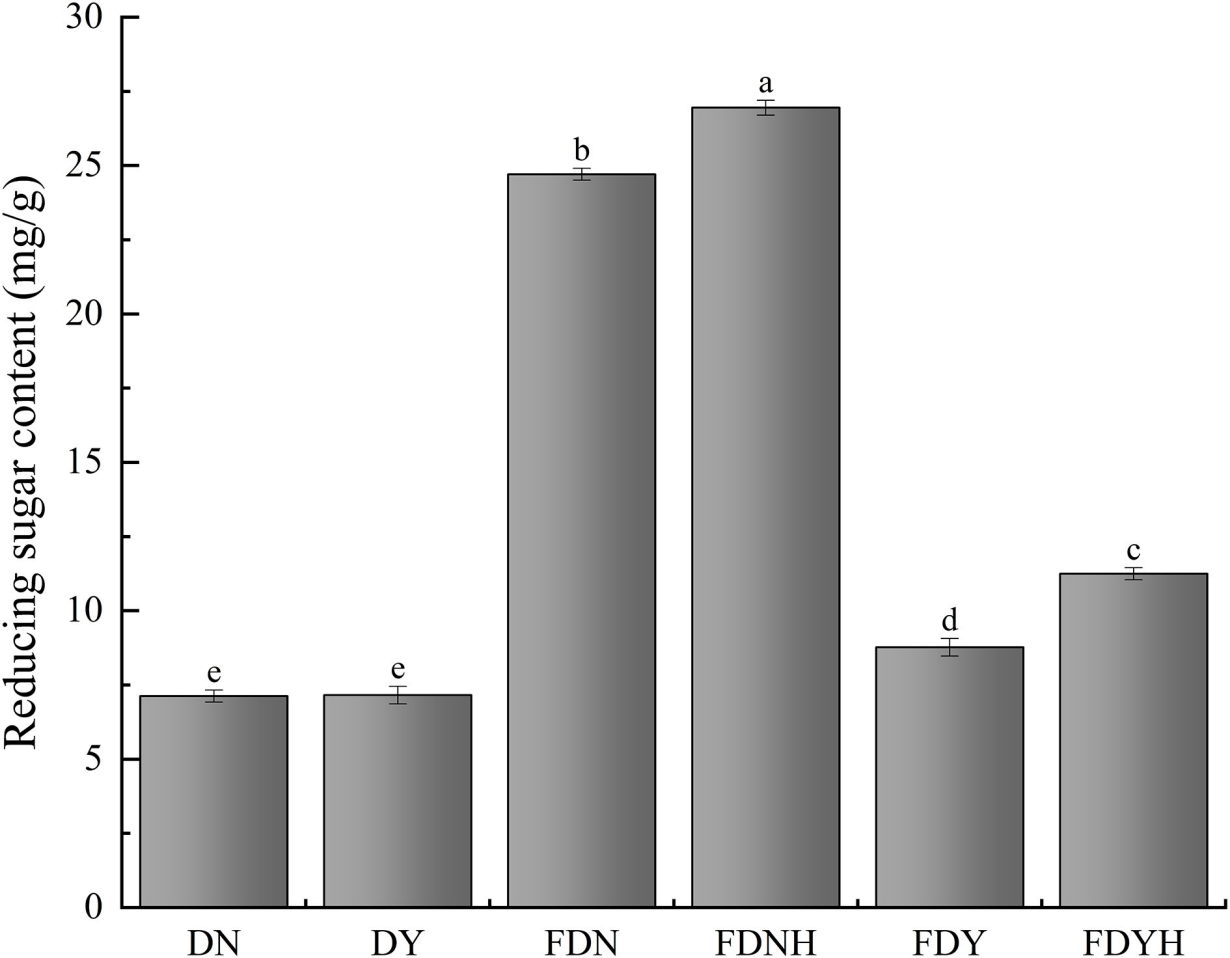
Reducing sugar content. Different letters indicate significant differences (*P* < 0.05). The error bars represent standard deviations.

As shown in Figure 1, reducing sugar contents of all fermented samples differed significantly (*P* < 0.05). Among them, DN (7.13 ± 0.2 mg/g) and DY (7.16 ± 0.3 mg/g) had significantly less reducing sugar content than other fermented samples. This may be the likely reason that high starch content (71–75%) of glutinous rice includes a high percentage of amylopectin (~99.70%) and a very low amount of amylose (Guo L. et al., 2015; Qiu et al., 2021). By comparing with FDN and FDY, the reducing sugar contents of FDNH and FDYH were increased by 4.46 and 13.53%, respectively, with the addition of CH. This observation could be explained by the fact that microorganisms secrete extracellular enzymes, including α-amylase, β-amylase, and glucosidase, which hydrolyze starch molecules to create glucoses and maltooligosaccharides (Tu et al., 2021). In addition, microbes also require an energy source to thrive during the fermentation. Therefore, upon addition of CH to glutinous rice dough samples, CH may have increased the LAB count, which consequently promoted the metabolic activities of microorganisms and increased reduced sugar consumption. This phenomenon was consistent with the results from the change in fermentation behaviors. In brief, the analysis of reducing sugar indicated that microorganisms break down polysaccharides more quickly than they consume reducing sugar, resulting in the accumulation of reducing sugar in the dough. Glutinous rice has high glycemic index, and its long-term consumption increases the risk of developing chronic diseases such as diabetes and obesity (Chen et al., 2010). The mechanism of regulating starch digestion will be the focus of our future studies.

### 3.3 Effects of CH on protein degradation

**Figure 2:**
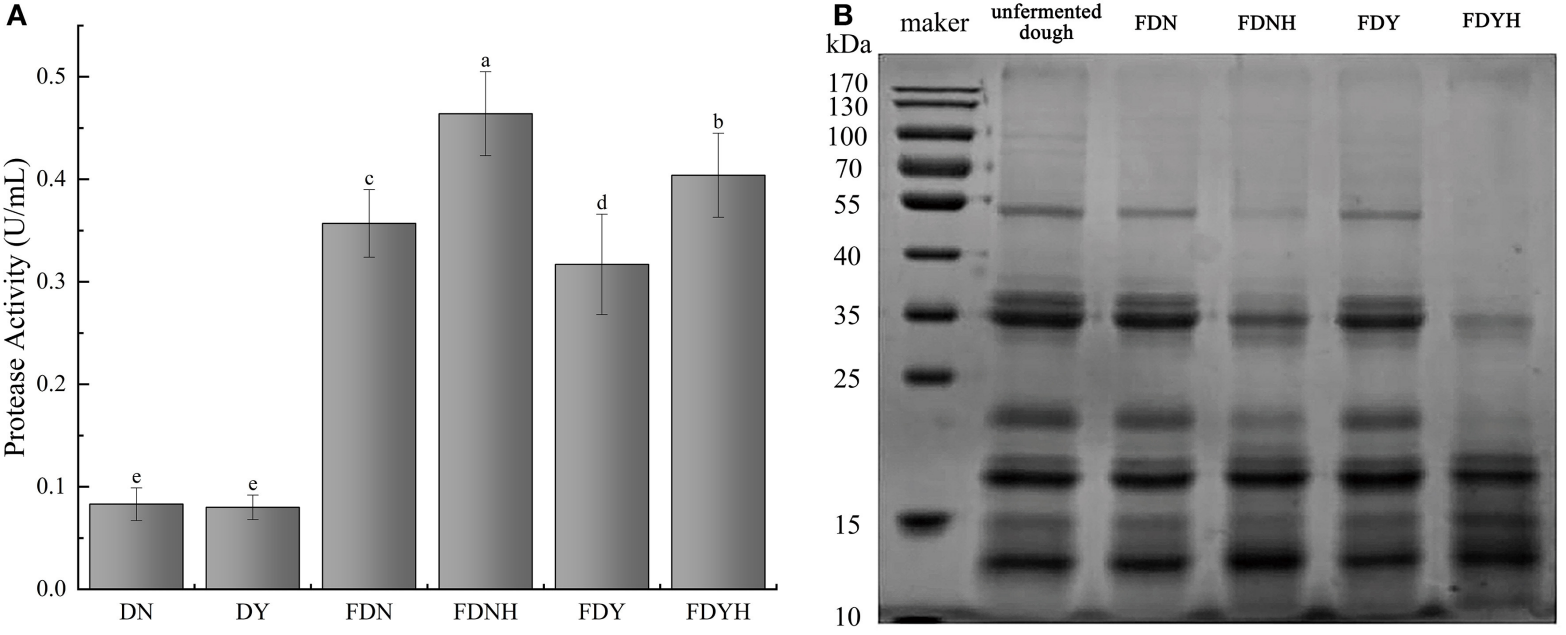
**(A)** Protease activity. Different letters indicate significant differences (*P* < 0.05). The error bars represent standard deviations. **(B)** Electrophoretic pattern of total protein by reducing SDS-PAGE in samples.

Rice has a protein content of 6.7–8.3%, which is only surpassed by reducing sugar. Protease activity of glutinous rice dough with or without CH treatment is presented in Figure 2A. Compared with unfermented samples, natural fermentation and yeast fermentation showed higher protease activity (*P* < 0.05) as fermentation progressed. Additionally, compared with the FDN and FDY, the protease activity of FDNH and FDYH, respectively, increased by 29.9 and 27.7%, which promoted the rate of protein degradation. Albumin, globulin, prolamin, and glutelin are the four primary protein types that make up rice protein. Among them, rice glutelin is a major protein source in rice, accounting for approximately 80% of total protein. SDS-PAGE analysis of glutinous rice dough samples is presented in Figure 2B. Previous studies have found that the primary protein subunits of glutelin by SDS-PAGE analysis were between 20, 33, and 53 kDa (Xu et al., 2016; Wang et al., 2019). Similar profiles were displayed by all samples (lanes 1–5), showing three main bands at 20, 35, and 55 kDa, respectively. Compared with UD, fermented glutinous rice dough samples showed lighter bands at 25–100 kDa and darker bands at 10–15 kDa. This banding patterns indicated that dough fermentation resulted in the breakdown of high molecular mass proteins. A distinct decrease in the intensity of the bands corresponding to molecular weights of ~20, 35, and 55 kDa was observed, especially in the yeast fermentation, when CH was added to the system. On the contrary, the bands at approximately 10–15 kDa became stronger. The gradual hydrolysis of glutinous rice proteins led to increases in polypeptide and amino acid contents. The findings further demonstrate that the addition of CH promoted protein degradation in glutinous rice dough.

Generally, protein degradation is attributable mainly to endogenous cereal proteases (Loponen et al., 2004; Dan et al., 2022). Owing to the acidic metabolites produced by the rapid growth of LAB during fermentation, the protein content of the dough samples degraded as a result of the cereal endogenous proteinases being activated by the low pH. The addition of CH improved the acidity of the system and resulted in higher endogenous protease activity. With the increase in protease concentration, microbial enzymes break down glutinous rice protein, converting it primarily into short peptides and even free amino acids, which serve as nitrogen sources for microbial development (Chen et al., 2020). Based on these results, the changes in the interaction between CH and endogenous protease that can influence the glutinous rice protein degradation were clarified. Therefore, it is reasonable to believe that CH significantly influenced the growth of LAB and increased the level of protease. Furthermore, CH also increased the amount of polypeptides and amino acids in fermented glutinous rice dough, enhancing the nutritional profile and boosting the nutritional value of the aforementioned goods.

### 3.4 Effects of CH on texture and microstructure

As shown in Table 2, with increasing fermentation time, the hardness of fermented samples decreased, and the adhesiveness, springiness, and gumminess increased. Less hardness and more springiness are the characteristics of a well-fermented glutinous rice dough. Compared with FDN and FDY, FDNH and FDYH have a lower hardness and higher adhesiveness, springiness, and gumminess, which indicated that CH played a role in connecting the starch granule gumminess. In the process of natural fermentation, the hardness of FDNH was significantly lower than that of FDN after 8 h, and the adhesiveness and springiness of FDNH were significantly higher than that of FDN, while no statistically significant change was observed in the gumminess of dough samples. After 1 h of yeast fermentation, the hardness of dough samples significantly decreased with the addition of casein, while its adhesiveness and springiness significantly increased. In contrast to FDY, the gumminess of FDYH significantly increased after 3 h of yeast fermentation. Related studies have shown that CH displayed beneficial functional properties in doughmaking, including low firmness and high volume (Kenny et al., 2000). On the one hand, the dough samples had more LAB with the addition of CH. As a result of the LAB metabolism, which increases lactic acid and protease activity and their need for nitrogen source for growth, the proteins in the dough samples started to break down. Protein breakdown improves the properties of the texture of dough. On the other hand, LAB break down the amylopectin in glutinous rice starch. This causes amylopectin's average degree of polymerization to decline and its average chain length to shorten. Since the amount of amylopectin polymerization is strongly correlated with glutinous rice dough hardness, fermentation makes the dough soft (Li et al., 2019). Similarly, Schmiele et al. (2015) discovered that soy protein hydrolysate weakened the structure of the gluten network, shortened the dough's stability time, and decreased its hardness by altering hydrogen bond, hydrophobic interaction, and covalent bond interactions. Selectively hydrolyzed soy protein conferred steamed bread with lower hardness and higher viscoelasticity (Li et al., 2021), while the addition of rice protein hydrolysate caused a fine bubble cell structure in the gluten-free rice starch bread crumb and improved the textural properties (Honda et al., 2021). Thus, it can be suggested that CH contributes to the texture of the glutinous rice dough.

**Table 2:** Textural properties of the samples.

**Texture properties**	**Samples**	**Fermentation time (natural fermentation)/h**						
		**0**	**2**	**4**	**6**	**8**	**10**	**12**
Hardness/g	SDN	170.29 ± 5.82^a, A^	158.44 ± 4.58^a, A^	142.90 ± 4.43^a, B^	137.17 ± 5.39^a, B^	109.42 ± 2.69^a, C^	100.85 ± 2.53^a, CD^	90.23 ± 2.62^a, D^
	SDNH	168.17 ± 6.44^a, A^	147.95 ± 5.07^a, A^	137.28 ± 2.49^a, B^	129.00 ± 8.00^a, B^	104.26 ± 1.07^b, C^	90.76 ± 2.90^b, CD^	82.13 ± 2.82^b, D^
Adhesiveness/(g·s)	SDN	6.58 ± 2.23^b, C^	8.51 ± 2.14^b, BC^	11.46 ± 5.88^b, BC^	15.11 ± 4.93^b, BC^	23.88 ± 8.52^b, AB^	32.65 ± 6.85^b, A^	39.59 ± 5.44^b, A^
	SDNH	19.83 ± 5.47^a, C^	21.22 ± 3.65^a, C^	23.31 ± 2.71^a, C^	32.44 ± 5.91^a, C^	51.30 ± 3.35^a, B^	59.20 ± 5.29^a, AB^	68.34 ± 4.29^a, A^
Springiness	SDN	0.28 ± 0.04^a, C^	0.31 ± 0.06^b, C^	0.34 ± 0.04^a, BC^	0.36 ± 0.09^b, BC^	0.43 ± 0.02^b, ABC^	0.49 ± 0.07^b, AB^	0.55 ± 0.05^b, A^
	SDNH	0.47 ± 0.21^a, C^	0.55 ± 0.13^a, BC^	0.64 ± 0.26^a, ABC^	0.74 ± 0.12^a, ABC^	0.88 ± 0.07^a, ABC^	0.95 ± 0.08^a, AB^	1.02 ± 0.05^a, A^
Gumminess/g	SDN	17.29 ± 2.78^a, C^	18.91 ± 2.88^a, BC^	20.04 ± 3.66^a, BC^	21.98 ± 2.69^a, BC^	24.46 ± 3.34^a, ABC^	27.66 ± 2.90^a, AB^	31.47 ± 3.16^a, A^
	SDNH	14.42 ± 1.99^a, D^	15.83 ± 2.14^a, D^	17.00 ± 3.37^a, D^	20.79 ± 2.98^a, CD^	25.16 ± 2.39^a, BC^	29.56 ± 1.96^a, AB^	33.75 ± 2.31^a, A^
**Texture properties**	**Samples**	**Fermentation time (yeast fermentation)/h**						
		**0**	**0.5**	**1**	**1.5**	**2**	**2.5**	**3**
Hardness/g	SDY	168.30 ± 7.09^a, A^	142.30 ± 6.59^a, B^	130.37 ± 7.62^b, BC^	122.65 ± 2.28^b, CD^	111.42 ± 3.78^b, DE^	100.85 ± 4.53^b, EF^	89.65 ± 1.13^b, F^
	SDYH	168.10 ± 4.38^a, A^	136.78 ± 1.98^a, B^	116.00 ± 3.85^a, C^	108.85 ± 3.69^a, C^	84.26 ± 2.07^a, D^	77.76 ± 1.87^a, D^	67.78 ± 2.16^a, E^
Adhesiveness/(g·s)	SDY	15.66 ± 1.93^a, D^	17.14 ± 1.08^a, CD^	18.80 ± 1.29^b, CD^	23.17 ± 1.07^b, BC^	25.21 ± 2.13^b, AB^	27.65 ± 3.85^b, AB^	29.25 ± 1.00^b, A^
	SDYH	15.46 ± 2.55^a, E^	23.31 ± 4.44^a, DE^	32.44 ± 2.50^a, CD^	42.39 ± 2.68^a, BC^	45.30 ± 1.68^a, AB^	50.20 ± 7.02^a, AB^	54.67 ± 3.92^a, A^
Springiness	SDY	0.34 ± 0.03^a, A^	0.33 ± 0.02^a, A^	0.38 ± 0.08^b, A^	0.43 ± 0.04^b, A^	0.44 ± 0.02^b, A^	0.45 ± 0.07^b, A^	0.47 ± 0.05^b, A^
	SDYH	0.35 ± 0.00^a, B^	0.64 ± 0.26^a, AB^	0.74 ± 0.12^a, A^	0.87 ± 0.06^a, A^	0.88 ± 0.07^a, A^	0.91 ± 0.08^a, A^	0.95 ± 0.03^a, A^
Gumminess/g	SDY	19.24 ± 1.99^a, B^	20.04 ± 3.66^a, B^	21.98 ± 2.69^a, AB^	22.31 ± 2.58^a, AB^	24.46 ± 3.34^a, AB^	26.66 ± 1.90^b, AB^	28.66 ± 0.90^b, A^
	SDYH	19.84 ± 1.18^a, D^	22.25 ± 3.29^a, CD^	24.04 ± 0.96^a, CD^	25.72 ± 2.23^a, BC^	27.36 ± 1.39^a, BC^	30.16 ± 0.58^a, AB^	33.22 ± 0.41^a, A^

Values are expressed as means ± standard deviation (n = 3). Different lowercase (or uppercase) letters in the same column (or row) indicated significant differences between groups (P < 0.05).

**Figure 3:**
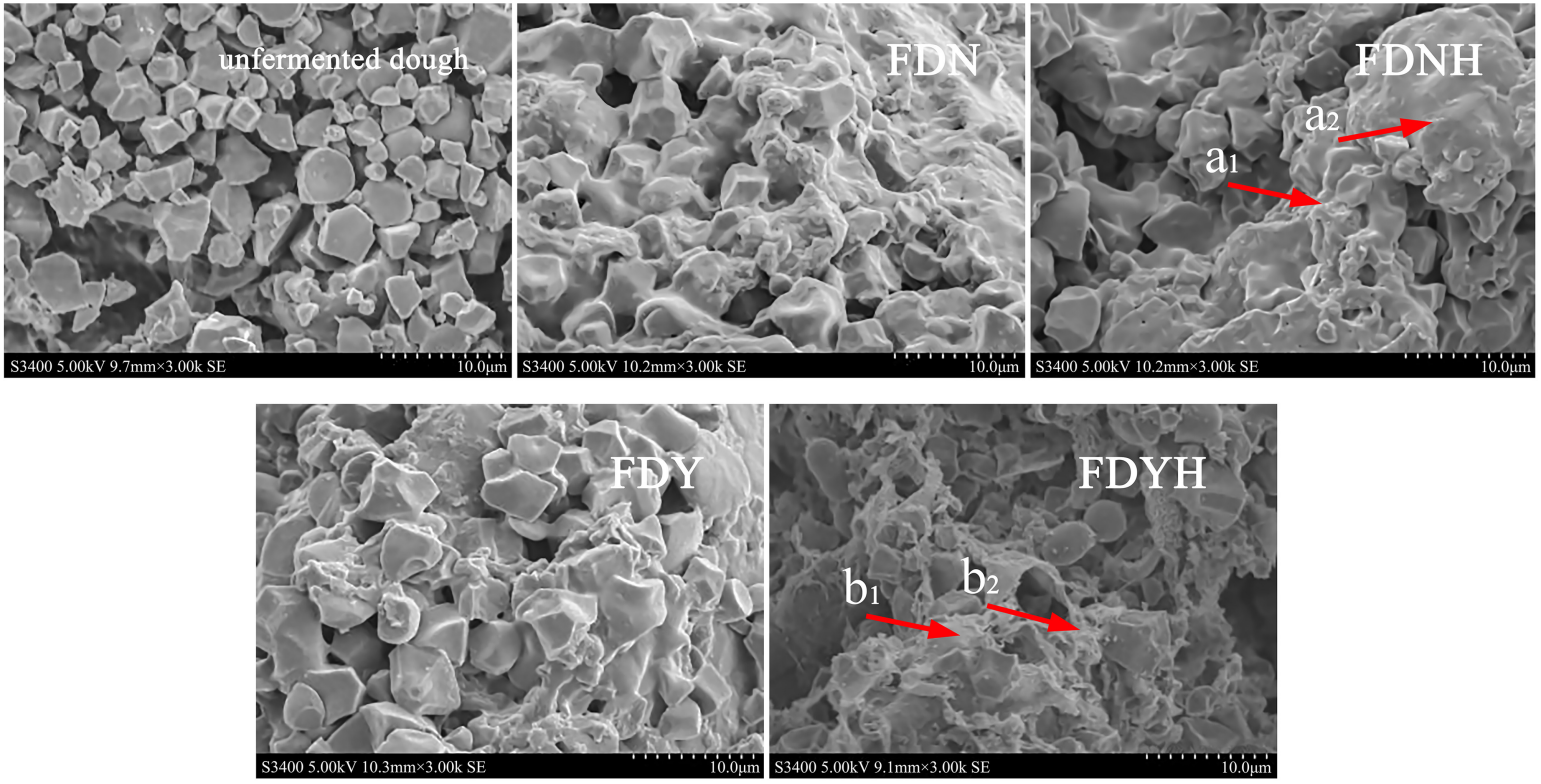
Scanning electron micrographs of dough samples. Different lower letters represent the changes in the microstructure of the dough after the addition of casein hydrolysate.

Previous studies have shown that the microstructure of dough was a continuous matrix, i.e., a gluten network. The starch granules incorporated into the gluten network made up the dough's microstructure to create a continuous film for wrapping CO_2_ gas (Chen et al., 2023). The major component of glutinous rice dough is starch and has a prominent effect on the quality of the final product. The effect of the addition of casein hydrolysates on the microstructure of glutinous rice dough is shown in Figure 3. The starch and protein particles in DN were unaltered, agglomerated, and displayed distinct flakes, but the gluten did not develop. With the progress of fermentation, the grain edge started to compartmentalize and show minor corrosion. After the addition of CH, the microstructure of dough showed edge regionalization and more serious corrosion in Figure 3 (arrow a_1_, a_2_, b_1_, and b_2_). The microstructure of glutinous rice dough samples is built on starch. The reason for the above phenomenon could be that amylase may have altered the structure of amylopectin and amylose, which, in turn, altered the network structure of starch, as well as other substances (proteins and lipids), explaining the above phenomenon (Meng and Kim, 2020a). CH treatment was confirmed to influence the microstructural properties of glutinous rice dough.

### 3.5 Effects of CH on the bacterial composition

High-throughput sequencing was utilized to examine the bacterial composition of the fermented dough samples (natural fermentation and yeast fermentation) in order to assess the impact of casein hydrolysates on the microflora of glutinous rice dough. A total of 1,129,755 effective tags were obtained from all dough samples after the quality control by Ion S5TMXL (Thermo Fisher Scientific, Shanghai, China), with an average of 62,764 tags per sample. The average of OTUs was 360 with 97% similarity. The Chao1, Ace, Shannon, and Simpson indices were used to evaluate and investigate the microbial diversity in dough samples (Table 3). The Chao1, ACE, and Shannon indices of the fermented dough samples (FDN and FDNH, FDY and FDYH) decreased in comparison to DN and DY, indicating that the richness and diversity of microorganisms had reduced. The relatively greater concentrations of dominant LAB (Table 1), which were helpful for the growth inhibition of contaminating, may help to explain this phenomenon (Dan et al., 2022; Wang et al., 2023). The microbial diversity of the dough samples gradually increased with the addition of CH, showing that CH provided more nitrogen sources, which led to an increase in the number of bacteria (Sharma et al., 2021).

**Table 3:** Alpha diversity of the samples.

**Samples**	**Natural fermentation**			
	**Chao1**	**ACE**	**Shannon**	**Simpson**
DN	312.10	321.87	2.94	0.78
SDN	210.32	221.45	2.58	0.69
SDNH	239.39	250.33	3.37	0.82
**Samples**	**Yeast fermentation**			
	**Chao1**	**ACE**	**Shannon**	**Simpson**
DY	386.71	394.60	3.43	0.81
SDY	273.83	284.44	2.78	0.77
SDYH	371.39	376.49	3.86	0.82

Values are the means ± standard deviations (n = 3).

**Figure 4:**
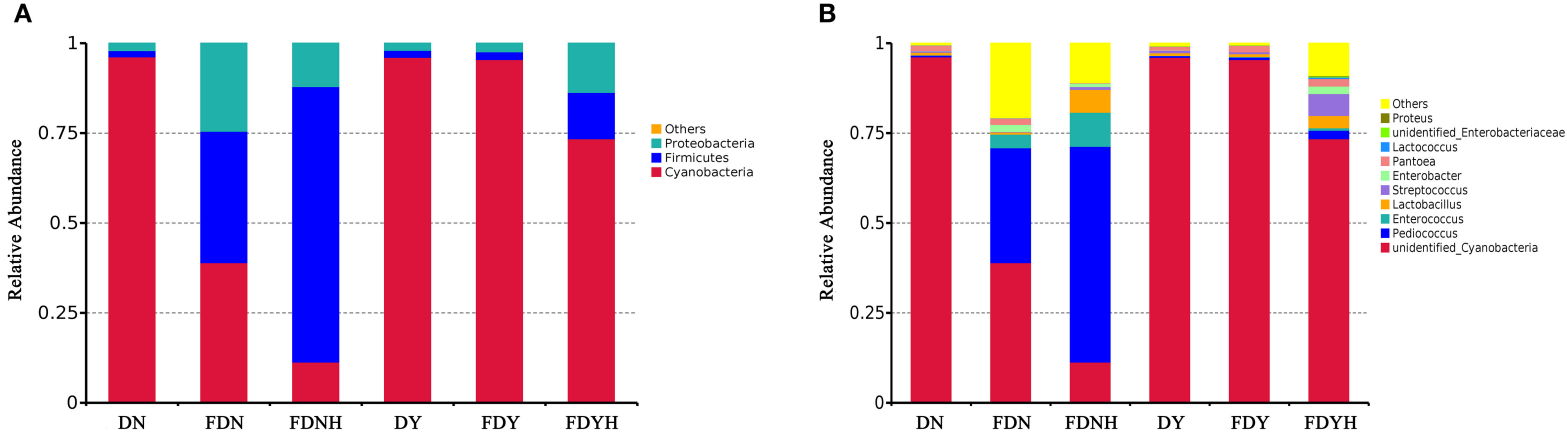
Relative abundances of bacterial community of dough samples at the phylum **(A)** and genus **(B)** levels.

The relative abundance distribution at the phylum and genus levels was shown to investigate the bacterial composition in glutinous rice doughs (Figure 4). At the phylum level, *Cyanobacteria, Firmicutes*, and *Proteobacteria* were the dominant bacterial communities in glutinous rice dough samples (Figure 4A). CH treatment improved the relative abundance of *Firmicutes* and *Proteobacteria* but reduced the relative abundance of *Cyanobacteria* in both natural and yeast fermentation. According to previous reports, *Firmicutes* are the most prevalent phylum of bacteria in fermented foods (Dan et al., 2022). In general, at the phylum level, the bacterial composition of glutinous rice dough samples is typically relatively straightforward. At the genus level, *Pediococcus, unidentified_Cyanobacteria, Enterococcus, Lactobacillus*, and *Streptococcus* were the top five bacterial genera in samples (Figure 4B). In comparison to FDN (32.01%) and FDY (0.66%), the CH treatment increased the abundance of *Pediococcus* in FDNH (59.35%) and FDYH (2.36%), respectively. This might be because CH offer more nitrogen sources, which is beneficial to the development of *Pediococcus* (Aspmo et al., 2005). Additionally, compared with FDNH (9.49%) and FDYH (0.66%), the abundance of *Enterococcus* was lower in FDN (3.74%) and FDY (0.13%). Djellouli et al. (2017) found that adding the seafood protein hydrolysates to the culture media stimulated the growth of *Enterococcus*. The addition of CH increased the abundance of *Lactobacillus* in samples by 10-fold and 4-fold while increasing the abundance of *Streptococcus* by 4- and 11-fold. LAB is regarded as one of the most important dough microorganisms because of its proteolytic activity, volatility, and antibacterial capability (Wang et al., 2020). CH increases the amount of LAB in dough, potentially enhancing the probiotic effects on glutinous rice dough and improving human health. As *Lactobacillus* populations rise as a result of CH treatment, fermented glutinous rice dough may experience greater probiotic advantages, which would then enhance human health. This is due to the fact that members of the Lactobacilli phylum frequently exhibit great probiotic qualities, antioxidant activities, and the capacity to decrease cholesterol (Guo C.-F. et al., 2015). Similarly, the growth of *L. plantarum* and *S. cerevisiae* in sourdough was aided by shrimp protein hydrolysates and amaranth-selective hydrolyzed protein (Karimi et al., 2020, 2021). In previous research studies, CH or milk protein hydrolysates were revealed to be promoting the growth of *Streptococcus* and thus reducing the fermentation time of yogurt or fermented milk (Lucas et al., 2004). Moreover, the abundance of *Enterobacter* genus decreased after the addition of CH. This result was in agreement with existing findings by Pei et al. (2017).

**Figure 5:**
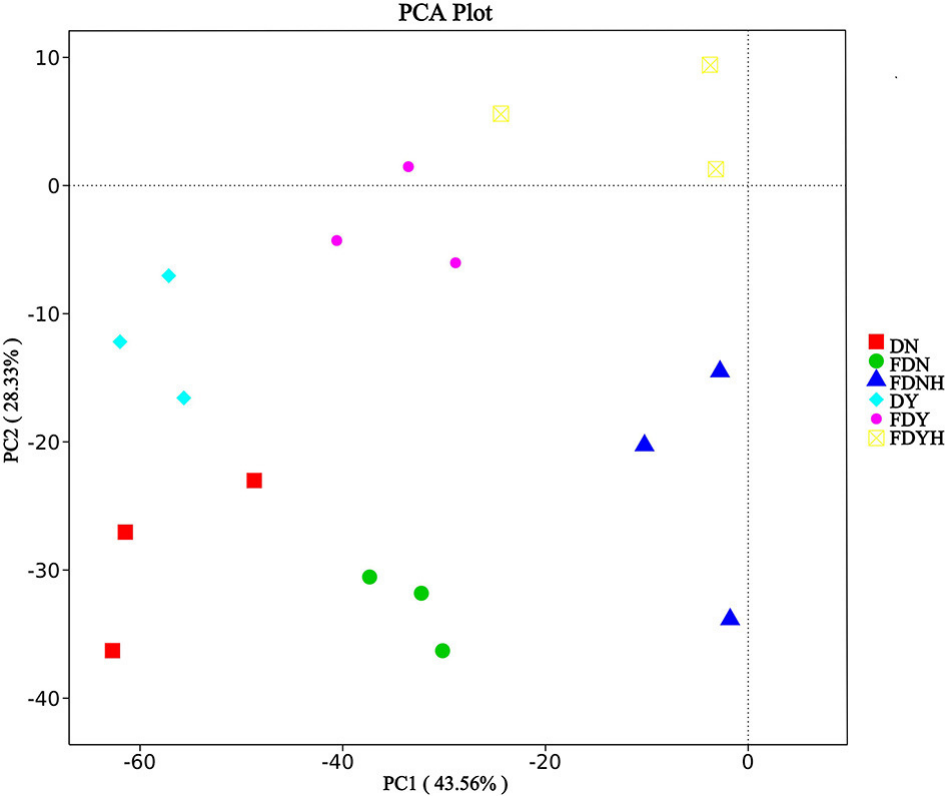
Principal Component Analysis (PCA) of bacterial communities. Different colors represent different samples.

The PCA result showed significant separation in bacterial communities among various samples (Figure 5). In total, 61.89% of variances of species were explained by the two axes (PC1 43.56% and PC2 28.33%). All of the dough samples clearly separated from one another, indicating their diversity of the bacterial communities. These results further confirmed that retarding CH for a long time allowed sufficient time for microbial metabolism, boosting formation, and the accumulation of flavor precursors and compounds in retarded sponge dough samples. Compared with naturally fermented samples, yeast fermented samples showed no significant differences in bacterial communities. This may be due to the fact that yeast can utilize casein for growth and that yeast and LAB compete with each other. Furthermore, yeast fermented samples ferment in a relatively short time, and the LAB colonies that contribute to the fermentation process are all wild bacteria that have not been tamed and have poor fermentation ability. As a result, the bacterial community in yeast-fermented dough did not significantly change.

**Figure 6:**
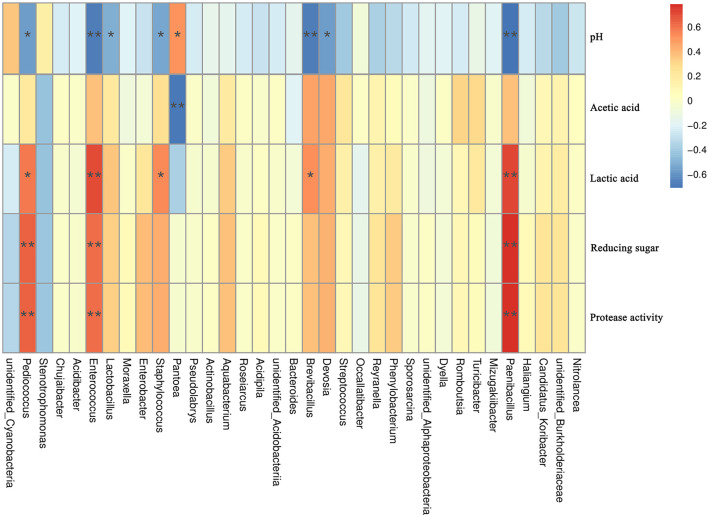
Correlation between dominant bacterial and environmental factors in all samples. The corresponding value of the intermediate heat map is the Spearman correlation coefficient with 1 indicating positive correlation (red) and −1 indicating negative correlation (blue). *Significant correlations (*P* < 0.05); **highly significant correlations (*P* < 0.01).

Environmental factors (pH, lactic acid, acetic acid, reducing sugar content, and protease activity) were used to analyze their Spearman correlation with the abundance of bacterial species in dough samples. The correlation results (Figure 6) showed that pH is significantly positively correlated with *Pantoea* (*R* = 0.51, *P* < 0.05). There are significant negative correlations between pH and seven species (*P* < 0.05), among which *Paenibacillus* (*R* = −0.71), *Brevibacillus* (*R* = −0.67), *Enterococcus* (*R* = −0.67), and pH have the highest negative correlations. The results indicated that, for the microorganisms listed above, pH was a crucial driving force. Acetic acid content presented a significant negative correlation with *Pantoea* (*R* = −0.69, *P* < 0.05), while there is no significant positive correlation with dominant bacteria. Lactic acid content showed a significantly positive correlation with five species (*P* < 0.05). *Paenibacillus* and *Enterococcus* exhibited the highest negative correlation with pH (*R* = 0.71). Reducing sugar content and protease activity showed significant positive correlations with three species (*P* < 0.05), and the R value of the correlations was >0.6.

## 4 Conclusion

This study analyzed the changes in the physicochemical properties and bacterial composition of glutinous rice dough produced by natural fermentation and yeast fermentation after the addition of CH. First, adding casein hydrolysates shortened fermentation by at least 2 h and increased the amount of LAB, lactic acid, and acetic acid in glutinous rice dough. CH enhanced protease activity and protein degradation while also increased the content of reducing sugar. Meanwhile, the texture of dough was made softer by the addition of CH. This indicated that CH can regulate the high hardness of glutinous rice dough. When investigating the effects of CH on the bacterial population of dough samples, we found that CH increased the bacterial richness and diversity of dough samples and stimulated the growth of *Pediococcus, Enterococcus, Lactobacillus*, and *Streptococcus*. Moreover, the Spearman correlation analysis showed that the abundance of some microorganisms was associated with environmental factors (pH, lactic acid, acetic acid, reducing sugar content, and protease activity). With the information, CH application can improve the fermentation properties of glutinous rice dough while also enhance its nutritional value. Additionally, CH will become more specialized and efficient as food additives for fermented grain products in future.

## Data availability statement

The original contributions presented in the study are included in the article/supplementary material, further inquiries can be directed to the corresponding authors.

## Author contributions

LG: Conceptualization, Funding acquisition, Investigation, Writing—original draft. BL: Conceptualization, Data curation, Formal analysis, Investigation, Methodology, Writing—original draft. YJ: Funding acquisition, Project administration, Resources, Validation, Writing—original draft. WZ: Investigation, Project administration, Resources, Validation, Writing—original draft. JH: Data curation, Formal analysis, Writing—original draft. WQ: Data curation, Formal analysis, Writing—original draft. YH: Investigation, Software, Validation, Writing—original draft. XZ: Formal analysis, Funding acquisition, Project administration, Resources, Writing—review & editing. XY: Formal analysis, Funding acquisition, Project administration, Resources, Writing—review & editing.
